# A rapid and high-throughput multiplex genetic detection assay for detection, semi-quantification and virulence genotyping of *Helicobacter pylori* in non-invasive oral samples

**DOI:** 10.3389/fcimb.2023.1267288

**Published:** 2023-09-29

**Authors:** Wenjing Chi, Su Wang, Tao Liu, Wenrong Jiang, Li Ding, Yingxin Miao, Feng Yang, Jinghao Zhang, Danian Ji, Zili Xiao, Haowei Zhu, Yong Wu, Zhijun Bao, Hu Zhao, Shiwen Wang

**Affiliations:** ^1^ Department of Laboratory Medicine, Huadong Hospital Affiliated to Fudan University, Shanghai, China; ^2^ Shanghai Key Laboratory of Clinical Geriatric Medicine, Huadong Hospital Affiliated to Fudan University, Shanghai, China; ^3^ Research Center on Aging and Medicine, Fudan University, Shanghai, China; ^4^ Department of Endoscopy, Huadong Hospital Affiliated to Fudan University, Shanghai, China; ^5^ Department of Research and Development, Ningbo HEALTH Gene Technologies Co., Ltd, Ningbo, China; ^6^ Department of Gerontology, Huadong Hospital Affiliated to Fudan University, Shanghai, China

**Keywords:** *Helicobacter pylori*, high-throughput multiplex genetic detection assay (HMGA), oral samples, performance evaluation, semi-quantification, virulence genes

## Abstract

**Aim:**

This study established a high-throughput multiplex genetic detection assay (HMGA) for rapid identification, semi-quantification and virulence analysis of *Helicobacter pylori* directly from the clinical non-invasive oral samples.

**Methods:**

The gastric mucosa and oral samples were collected from 242 patients in Shanghai from 2021 to 2022. All the samples were detected by routine clinical tests for *H. pylori* and Sanger sequenced for inconsistent results. A new multiplex PCR assay providing results within 4 hours was designed and optimized involving fluorescent dye-labeled specific primers targeted *16S rRNA* gene, semi-quantitative gene *ureC* and 10 virulence genes of *H. pylori*. Semi-quantification was carried out by simulating the serial 10-fold dilutions of positive oral samples, and the *H. pylori* loads in different clinical samples were further compared. The mixed plasmids of virulence genes *vacA s1*, *vacA m1* and *vacA m2* were used to evaluate the performance on different genotypes. The consistency of 10 virulence genes in gastric mucosa, saliva, mouthwash and dental plaque of *H. pylori*-positive patients was compared.

**Results:**

The non-invasive HMGA was highly specific for detection of all 12 targets of *H. pylori* and human internal reference gene *β-globin*, and the sensitivity to all target genes could reach 10 copies/μL. Compared with routine clinical tests and sequencing, non-invasive HMGA has a high level (>0.98) of sensitivity, specificity, accuracy, PPV, NPV and kappa coefficient for direct detection of *H. pylori* in oral samples. Moreover, by detecting peak area levels of *ureC*, it was confirmed that the *H. pylori* loads in gastric mucosa were significantly higher than those of the three kinds of oral samples (*p*<0.05). We also found that 45.0% (91/202) of patients had different *H. pylori* virulence genes in different oral samples. The concordance of positive detection rates of each virulence gene between saliva and gastric mucosa was more than 78% (*p*<0.05).

**Conclusion:**

The non-invasive HMGA proved to be a reliable method for the rapid *H. pylori* identification, semi-quantification and detection of 10 virulence genes directly in oral samples, providing a new idea for non-invasive detection of *H. pylori*.

## 1 Introduction

*Helicobacter pylori* is one of the most common causes of cancer-related deaths and is thought to infect half of the world’s population (Kesharwani et al., 2023). The common clinical detection methods of *H. pylori* are Urea breath test (non-invasive test), histochemical method and culture (invasive test), all of which have certain advantages and disadvantages (Kismat et al., 2022). ^13^C/^14^C-urea breath test, as the gold standard for *H. pylori* detection, is mainly applicable to screen *H. pylori* in the stomach during physical examination. Although there is no physical pain to the patients, its results may be affected by drugs and endogenous production of CO_2_ depending much on basal metabolic rate (Eisdorfer et al., 2018; Chen et al., 2023). Histochemistry has high sensitivity and specificity, but the operation is complicated and brings greater pain to patients due to gastroscopy biopsy (Kocsmár and Lotz, 2022). Even worse, the harsh culture conditions of *H. pylori* result in a low positive detection rate (McNulty, 2023).

The oral cavity is an important storage location for microbial population and a potential reservoir of *H. pylori*, which may cause the recurrence of *H. pylori* infection in the stomach (Nagata et al., 2023). In addition, it has been reported that the *H. pylori* in the oral cavity is highly homologous to that in stomach and closely related to the occurrence and development of gastric diseases(Siavoshi et al., 2005; Chen et al., 2022). However, some researchers have indicated that the genotypes of *H. pylori* in the oral cavity and stomach are different (Sohrabi et al., 2021). Therefore, the correlation between oral and gastric *H. pylori* infection is controversial (Mao et al., 2021). Moreover, the positive detection rates of *H. pylori* infection in oral samples vary greatly among different detection methods, ranging from 0% to 100% (Wang et al., 2014; Zhou et al., 2016; Xu et al., 2018). Therefore, there is an urgent need to develop a non-invasive detection technology for *H. pylori* in oral samples, providing a reliable etiological diagnosis basis for *H. pylori* infection.

Our team has previously established the HMGS platform for direct detection of *H. pylori* in gastric mucosa (Zhang et al., 2016a; Zhang et al., 2016b; Zhou et al., 2016). However, sampling methods have drawbacks such as invasion, complex sampling process and are only applicable to specialized laboratories. In the present study, we established a non-invasive detection technology with high accuracy, specificity and sensitivity for *H. pylori* in oral samples. In gastric mucosa, culture, histology, rapid urease test (RUT) and quantitative real-time PCR (qRT-PCR) were used as the comparators for the non-invasive HMGA platform. In oral samples, only RUT and qRT-PCR were used as the comparators for the non-invasive HMGA platform. And we further explored the consistency and difference between oral and gastric *H. pylori*, providing reliable etiological diagnosis basis for clinically rapid and convenient detection of *H. pylori*.

## 2 Methods

### 2.1 Ethics statement

This study was conducted in accordance with the Declaration of Helsinki and approved by Medical Ethics Committee of Huadong Hospital Affiliated to Fudan University. All participants have signed the informed consent forms (The Ethics Approval Number: 2020K080).

### 2.2 Inclusion & exclusion criteria

Patients who underwent upper gastrointestinal endoscopy due to recurrent upper gastrointestinal symptoms of abdominal pain, acid reflux, vomiting or abdominal distention, and chronic gastritis, peptic ulcer or gastric cancer with family history, were included(Kotilea et al., 2023). Patients who received anti-*H. pylori* therapy within 4 weeks preceding the study, experienced subtotal gastrostomy, suffered from severe cardiovascular and respiratory diseases or recently consumed NSAID or alcohol, were excluded(Kotilea et al., 2023). In addition, all included patients did not brush their teeth or drink water in the morning.

### 2.3 Collection of gastric specimens and oral specimens

From February 2021 to January 2022, 242 patients with oral samples and gastric mucosa who met the inclusion criteria and received gastroscopy in Huadong Hospital Affiliated to Fudan University were recruited into the final cohort. The gastric biopsy specimens were taken from two parts of antrum of the patients undergoing endoscopy. The two biopsies were homogenized, combined together, and then divided into four portions for the following tests: culture, RUT, qRT-PCR and non-invasive HMGA. Among 242 patients, there were 126 (52.1%, 126/242) males with mean age of 48.0 ± 18.8 years (age range: 15-93 years) and 116 (47.9%, 116/242) female patients (age range: 14-89 years, mean age: 50.0 ± 19.0 years). In addition, to further verify no interference in target genes, oral samples were obtained from 30 healthy volunteers.

The oral samples including saliva, mouthwash and dental plaque were collected. Firstly, 1 mL of the saliva from patients who did not drink, eat or brush teeth were collected into a disposable sterile sputum cup. Then, patients drink 1mL of 0.9% saline to rinse their mouths for 1 minute. The collected mouthwash was placed in a disposable sterile sputum cup. We then used the disposable probe to scrape the subgingival plaque from the patient’s first and second molars gently to avoid gums bleeding and transferred the scraped plaque into a sterile tube containing 1 mL of 0.9% saline.

**Figure 1:**
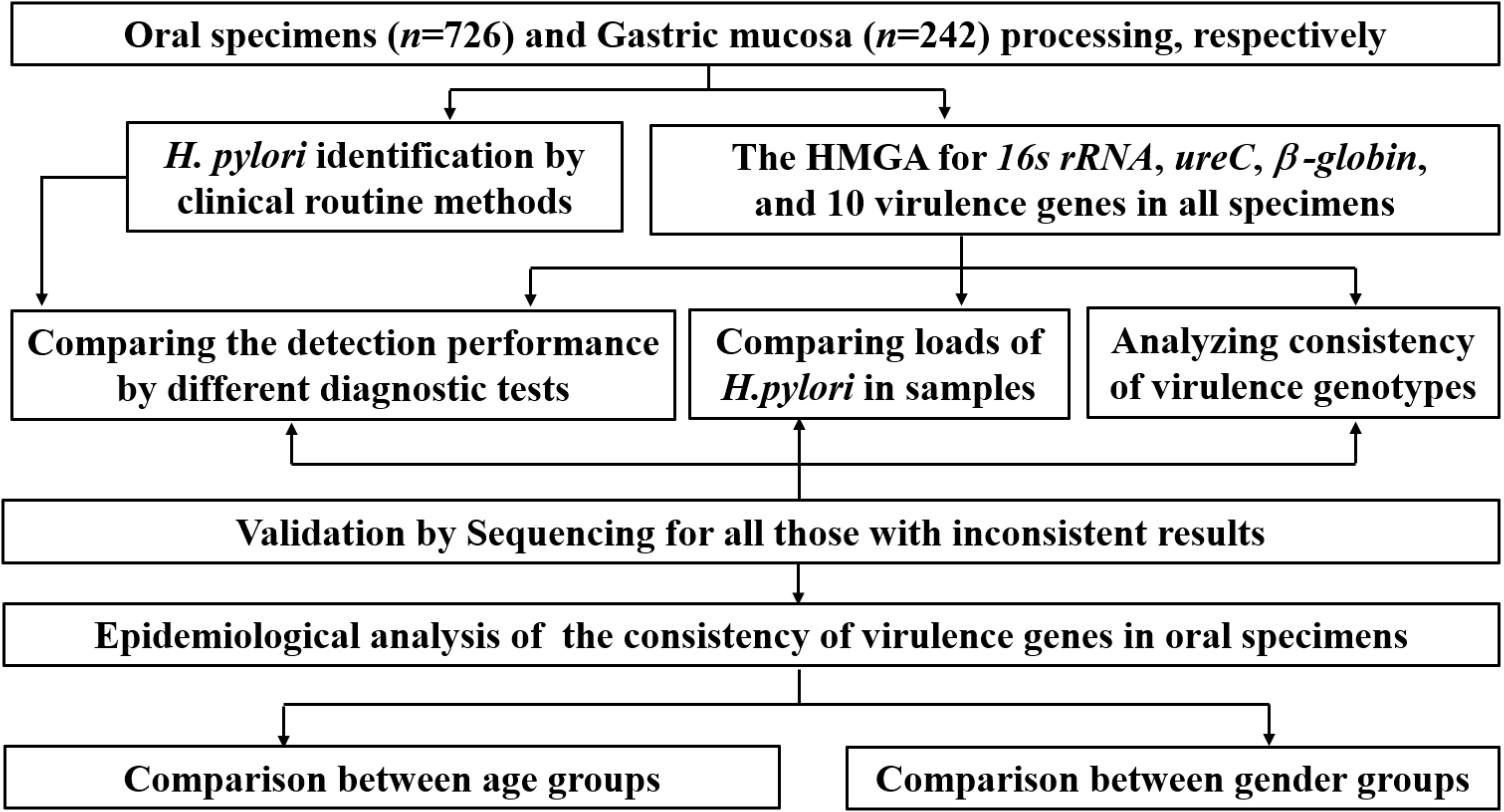
Workflow for oral specimens (n=726) and gastric mucosa specimens (n=242) processing.

All samples were stored for clinical performance verification, comparison of *H. pylori* loads and consistency analysis of virulence genes. The main experimental and analyzing procedure is shown in **Figure 1**. Firstly, the non-invasive HMGA was used to detect *H. pylori* in clinical samples, and further compare the detection performance of different diagnostic methods. Then, the differences of *H. pylori* loads in clinical samples were compared. And epidemiological characteristics of the consistency of virulence genes in oral samples were further analyzed. In addition, the consistency of virulence genotypes was compared between gastric mucosa and oral samples.

### 2.4 Clinical routine methods

Gastric mucosa specimens for histological examination were fixed in 4.5% buffered formalin and embedded in paraffin. A modified Giemsa stain was used to detect *H. pylori*. An experienced histopathologist reviewed each biopsy without knowing clinical data or *H. pylori* status. The gastric biopsies sent to the microbiology laboratory were homogenized and then inoculated on Columbia agar medium (OXOID Microbiology Products, Thermo Fisher Scientific Inc., MA, USA) containing 8% sterile defibrinated sheep blood and 0.5% selective antibiotics supplement. The cultures were kept at 35°C under microaerophilic conditions of 5% O_2_, 10% CO_2_ and 85% N_2_ for 3-7 days. Colonies with typical *H. pylori* morphology were selected and identified by Gram staining, RUT, oxidase and catalase tests. Notably, RUT was used to detect *H. pylori* in each oral and gastric biopsy specimen according to the instructions (*H. pylori* rapid detection kit, Huiyi Biology Companies, Shanghai, China). The brief operation was as follow. The gastric mucosa biopsy and oral samples were placed into semisolid 2% agar with a sterile needle and then incubated at room temperature. It was positive for *H. pylori* when the indicator light changes from yellow to red after 30min. The operation was performed as the instruction (Siavoshi et al., 2015). Moreover, qRT-PCR performed by *H. pylori* Nucleic Acid Detection Kit (Da’an Gene Co., Ltd, Guangzhou, China) was used to identify *H. pylori* in each sample by ABI Prism^®^7500 Real‐Time PCR Instrument (Thermo-Fisher, MA, USA). Specific primers and fluorescent probes were designed with the highly conserved region of *ureA* of *H. pylori* as the target. The CT value ≤ 27.02 indicated a positive *H. pylori* infection status.

### 2.5 Establishment of the non-invasive HMGA

#### 2.5.1 Primer design

The gene sequences of *H. pylori* strain-specific identification gene *16S rRNA*, single-copy semi-quantitative gene *ureC*, 10 virulence genes (*vacA s1*, *vacA s2*, *vacA m2*, *vacA m1*, *cagA*, *oipA*, *luxS*, *iceA2*, *iceA1* and *dupA*) and one human internal reference gene *β-globin* were downloaded from the National Center of Biotechnology Information (NCBI). Vector NTI (Invitrogen, Carlsbad, USA) was used for multiple sequence alignment, and highly conserved specific sequences were selected to design primers (Sun et al., 2021). Next, primers were designed to amplify each of the highly conserved regions using DNASTAR (DNASTAR Inc., Madison, WI, USA) and Primer Premier 6.0 (Premier Biosoft International, Palo Alto, CA, USA)(Sun et al., 2021). The primers are detailed in **Table 1**.

**Table 1:** The primer sequences and amplicon sizes of 13 target genes for non-invasive HMGA.

Types	Targets	Sequence (5’-3’)	Size (bp)	Concentration (nM)
Semi-Quantification	*ureC*	F: GTTCATGAAAGATTTCTTCAATCGCT	103	300
		R: TCAGCCACAACCCTTTTGAAGA		
Identification	*16S rRNA*	F: GTCTGGAGAGACTAAGCCCTCC	114	300
		R: TCATTACTGAAGCTGATTGCGC		
Virulence	*vacA m2*	F: GTGCAAGCATGGATTATGGTAARGA	130	300
		R: TCTTGCTTGATGGCCTGCATT		
	*iceA2*	F: GTTGTARTTAAAGTCGTTAATGGCAA	135	300
		R: TCACTTTACCCTTTGATGTGGTTAC		
	*dupA*	F: GTACCTATATCGCTAACGCRCT	152	400
		R: TCGTGGGGTADATAATCACTTGAGA		
	*cagA*	F: GTCAAAAATCCTRCCAAAAAGAATCAGT	174	400
		R: TCGATCKTTTTGAWGGGACACC		
	*iceA1*	F: GTGGCAAYTCTGAAAACACTCA	198	300
		R: TCCCAGGAATTTTTSTTGCRTCAA		
	*luxS*	F: GTCCCATAGGCGACCAATCCAY	205	300
		R: TCACACCAAAGTCAAAGCCCCT		
	*vacA m1*	F: GTAAATTGGCTATAATCCATGACYG	245	400
		R: TCGTTTRGAAACTGGCACYAGGTCAA		
	*vacA s1*	F: GTATGGAAWTACAACAAACACACC	263	300
		R: TCCTGCTTGAATGCGCCAAAC		
	*oipA*	F: GTATTATAGGGTTTAGGCACTCTCTT	281	300
		R: TCCCAATCACAAGCCCTGAAGAT		
	*vacA s2*	F: GTATGGAAWTACAACAAACACACC	290	300
		R: TCCTGCTTGAATGCGCCAAAC		
Internal Control	*β-globin*	F: GTAGAAAGCGAGCTTAGTGATACT	164	100
		R: TCCTCTTATCTTCCTCCCACAGCT		

F, forward primes; R, reverse primes.

#### 2.5.2 DNA extraction and multiplex PCR

Total DNA from the strains and clinical samples was extracted by an automatic nucleic acid extraction instrument with magnetic beads (Smart LabAssist, Taiwan Advanced Nanotech, Taiwan, China). Concentration of each extracted DNA was determined using the Nanodrop spectrophotometer (Thermo Fisher Scientific Inc., MA, USA). DNA was kept in -20°C for further analysis.

Multiplex PCR mixture with the 15µL final volume included 6µL of 2.5*NuHi SU11 PCR Mix (NuHigh Biotechnologies Co., Ltd, Suzhou, China), 1.5µL of pooled primers, 2.5µL of ddH_2_O, and 5µL of template. The primer pool consisted of one pair of primer for identification gene, one pair for semi-quantitation gene, ten pairs for virulence genes and one pair for the quality control gene. The primers were mixed in different proportions to achieve optimum sensitivity for all targets, and the final concentration of each primer in the pool was listed in **Table 1**. The PCR was performed using the Veriti 96-well Thermal Cycler (Applied Biosystems, California, USA): 50°C for 5 min; 95°C for 10 min; 32 cycles of 95°C for 30 seconds, 60°C for 30 seconds and 72°C for 1 min; and 72°C for 15 min.

#### 2.5.3 Capillary electrophoresis and fragment analysis

**Figure 2:**
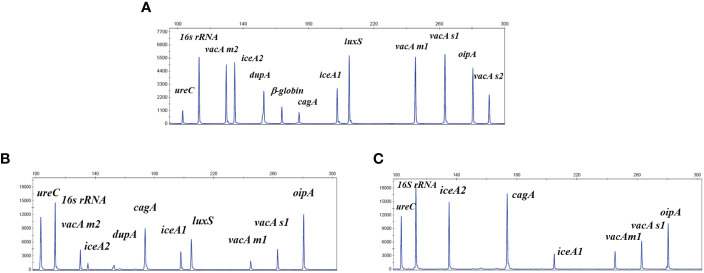
The non-invasive HMGA could produce specific amplification signals for 13 targets. The horizontal coordinate indicated the actual PCR product size(bp), and the vertical coordinate indicated the dye signal (rfu). **(A)** The specific amplification signals arising from the mixed plasmids of the 12 target genes of *H. pylori* and one *β*-globin gene at the same concentration of 20 copies/μL and 7 negative control pathogens DNA all at the concentration of 10^5^ copies/μL. **(B, C)** The specific amplification signals arising from the amplification of the traditional clinical isolates. Note that all gene targets were specifically amplified without non-specific amplification by the non-invasive HMGA.

The separation by capillary electrophoresis and fragment analysis using the Applied Biosystems 3500DX Genetic Analysis System (Applied Biosystems, California, USA) were same as our previous study (Sun et al., 2021). In this study, when the detection peak height of the target gene product fragment >500 relative fluorescence units (rfu), it was judged positive for the target gene (**Figure 2A**). The ddH_2_0 and seven negative control pathogens, including *Candida albicans* (*C. albicans*), *Streptococcus salivarius* (*S. salivarius*), *Acinetobacter baumannii* (*A. baumannii*), *Staphylococcus aureus* (*S.aureus*), *Pseudomonas aeruginosa* (*P. aeruginosa*), *Escherichia coli* (*E.coli*) and *Klebsiella pneumoniae* (*K. pneumoniae*) were used as negative quality controls throughout the detection. All results from the non-invasive HMGA were further verified by Sanger sequencing, as the gold standard for gene identification in this study (Drevinek et al., 2023). The identification gene for *H. pylori* with Sanger sequencing is 576 bp*-16S rRNA* referred to previously reported paper (Forward primer: (5′-TCTAACGAATAAGCACCGGCTA-3′), Reverse primer: (5′-GTGCAGCACCTGTTTTCAAGG-3′)(Hu et al., 2016).

#### 2.5.4 Optimization of the non-invasive HMGA

The 13 targets detection for the non-invasive high-throughput multiplex genetic detection assay (HMGA) were optimized as following principle: primer sequences were optimized so that each signature of detection target could be amplified specifically without cross-interaction; other reaction parameters of PCR components and procedures, such as buffer, enzyme, and reaction time, were also systematically optimized. Additionally, detections of *β-globin* in the clinical samples indicated that no significant nucleic acid degradation had occurred during specimen handling/storage. In addition, we mixed the plasmids of the 12 target genes of *H. pylori* and *β-globin* at the same concentration of 20 copies/μL and added seven negative control pathogen DNA, including *Candida albicans*, *Streptococcus salivarius*, *Acinetobacter baumannii*, *Staphylococcus aureus*, *Pseudomonas aeruginosa*, *Escherichia coli* and *Klebsiella pneumoniae* all at the concentration of 10^5^ copies/μL. The *H. pylori* negative results were validated in saliva (*n*=30), mouthwash (*n*=30) and dental plaque samples (*n*=30) from healthy people using RUT and qRT-PCR. Then the non-invasive HMGA was used to detect the extracted DNA of *H. pylori* negative oral samples from healthy people, respectively.

### 2.6 Evaluation of the semi-quantification of non-invasive HMGA

In order to prove whether non-invasive HMGA can semi-quantitatively detect *H. pylori*, the single copy gene *ureC* of *H. pylori* was selected as the detection target. Firstly, the standard strain ATCC26695 was fully mixed with the saliva of healthy volunteers to extract DNA from the simulated *H. pylori* positive saliva. The concentration of *ureC* in the standard strain was quantified by Droplet Digital PCR (dd-PCR), and the *ureC* gene was gradually diluted to 1×10^4^, 1×10^3^, 1×10^2^, 1×10^1^ and 1×10^0^ copies/μL, respectively. The non-invasive HMGA was used to detect the *ureC* gene at each gradient concentration for three times. The gradient dilution concentration of *H. pylori* was used as the horizontal coordinate, and the peak areas of the three assays corresponding to different gradient concentrations were as the vertical coordinate.

### 2.7 Statistical analysis

The true positive is defined as consistent positive results of both the detection methods and the gold standard. The true negative is defined as consistent negative results of both the detection methods and the gold standard. GraphPad Prism 8.0.2 (San Diego, CA, USA) was used for statistical analysis. The positive rates of *H. pylori* virulence genes from saliva, mouthwash and dental plaque samples in genders and ages were compared by the Chi-squared test. The Wilcoxon rank sum test was used for comparison of *ureC* peak area levels in different samples and above. For all figures, * means *p <*0.05, ** means *p <*0.01, *** means *p <*0.001, **** means *p <*0.0001, and n.s. means not significant (*p >*0.05). Differences were considered statistically significant for *p <*0.05.

## 3 Results

### 3.1 The non-invasive HMGA is specific for *H. pylori* identification

The results showed that 13 target genes had specific peaks without non-specific products amplified by their corresponding primers. Subsequently, As shown in **Figure 2A**, 13 specific amplification peaks were observed clearly. Two traditional clinical isolates were used to evaluate the accuracy of simultaneous detection of target genes of the non-invasive HMGA. All the specific amplification signals were observed clearly (**Figures 2B, C**). In addition, seven negative control pathogens did not produce any specific amplification peaks (**Supplementary Figures S2A–G**). As shown, only *β*-globin was found in the electrophoresis maps of saliva (**Supplementary Figure S2H**), mouthwash (**Supplementary Figure S2I**) and dental plaque (**Supplementary Figure S2J**), indicating that the detection of *H. pylori* target genes by non-invasive HMGA was not interfered by complex microorganisms in oral samples. The above results demonstrated that non-invasive HMGA was highly specific to all targets. Additionally, the minimum detection limit of non-invasive HMGA was as low as 10 copies/μL for simultaneous detection of 12 target genes using serial 10-fold dilution.

### 3.2 Positive detection rate and performance evaluation of non-invasive HMGA for *H. pylori* in oral samples

The results of non-invasive HMGA showed that *H. pylori* was detected in 208 (86.0%, 208/242) gastric mucosa, 194 (80.2%, 194/242) saliva samples, 167 (69.0%, 167/242) mouthwash samples and 128 (52.9%, 128/242) dental plaque samples. Furthermore, 202 patients (83.5%, 202/242) tested positive for more than one kind of oral samples, 169 patients (69.8%, 169/242) tested positive for more than two kinds of oral samples, and 115 patients (47.5%, 115/242) tested positive for all three oral samples. Additionally, the results of non-invasive HMGA showed that *H. pylori* was all negative in oral samples from 30 healthy volunteers.

Comparing with sequencing, the non-invasive HMGA system for detecting *H. pylori* in gastric mucosa (**Table 2**) and oral samples (**Table 3**) was evaluated. The sensitivities of non-invasive HMGA to *H. pylori* in saliva, gargle and dental plaque samples were 1.00, 1.00 and 0.99. respectively. The specificities of non-invasive HMGA detections were all 1.00 in oral samples. The kappa values measuring the consistency values of non-invasive HMGA and sequencing in saliva, mouthwash and dental plaque samples were 1.000, 1.000 and 0.992, respectively (**Tables 2**, **3**).

**Table 2:** Non-invasive HMGA exhibited high levels of accuracy for the identification of *H. pylori* in gastric biopsies when compared to conventional methods.

Methods		Sequencing		Sensitivity	Specificity	PPV	NPV	Accuracy	Kappa
		+	-						
Gastric mucosa									
Culture	**+**	56	0	0.269	1.000	1.000	0.183	0.372	0.197
	**-**	152	34						
Histology	**+**	205	0	0.976	1.000	1.000	0.872	0.980	0.920
	**-**	5	34						
Urease	**+**	184	10	0.885	0.706	0.948	0.500	0.860	0.504
	**-**	24	24						
qPCR	**+**	206	0	0.990	1.000	1.000	0.944	0.992	0.967
	**-**	2	34						
HMGA	**+**	208	0	1.000	1.000	1.000	1.000	1.000	1.000
	**-**	0	34						

+, positive for H. pylori; **-**, negative for H. pylori.

Kappa coefficient consistency test scoring criteria:

0.0~0.20: slight consistency; 0.21~0.40: fair consistency; 0.41~0.60: moderate consistency; 0.61~0.80: substantial consistency; 0.81~1: almost perfect consistency.

**Table 3:** Non-invasive HMGA exhibited high levels of accuracy for the identification of *H. pylori* in oral specimens when compared to conventional methods.

Methods		Sequencing		Sensitivity	Specificity	PPV	NPV	Accuracy	Kappa
		+	-						
Saliva									
Urease	**+**	168	23	0.866	0.521	0.880	0.490	0.798	0.378
	**-**	26	25						
qPCR	**+**	174	0	0.897	1.000	1.000	0.706	0.917	0.775
	**-**	20	48						
HMGA	**+**	194	0	1.000	1.000	1.000	1.000	1.000	1.000
	**-**	0	48						
Mouthwash									
Urease	**+**	119	38	0.730	0.519	0.758	0.482	0.661	0.244
	**-**	44	41						
qPCR	**+**	142	0	0.871	1.000	1.000	0.790	0.913	0.815
	**-**	21	79						
HMGA	**+**	162	0	0.994	1.000	1.000	0.988	0.996	0.991
	**-**	1	79						
Dental plaque									
Urease	**+**	78	55	0.605	0.513	0.586	0.532	0.562	0.118
	**-**	51	58						
qPCR	**+**	111	0	0.860	1.000	1.000	0.863	0.926	0.852
	**-**	18	113						
HMGA	**+**	128	0	0.992	1.000	1.000	0.991	0.996	0.992
	**-**	1	113						

### 3.3 Relative quantitative analysis of *H. pylori* infection in oral samples

**Figure 3:**
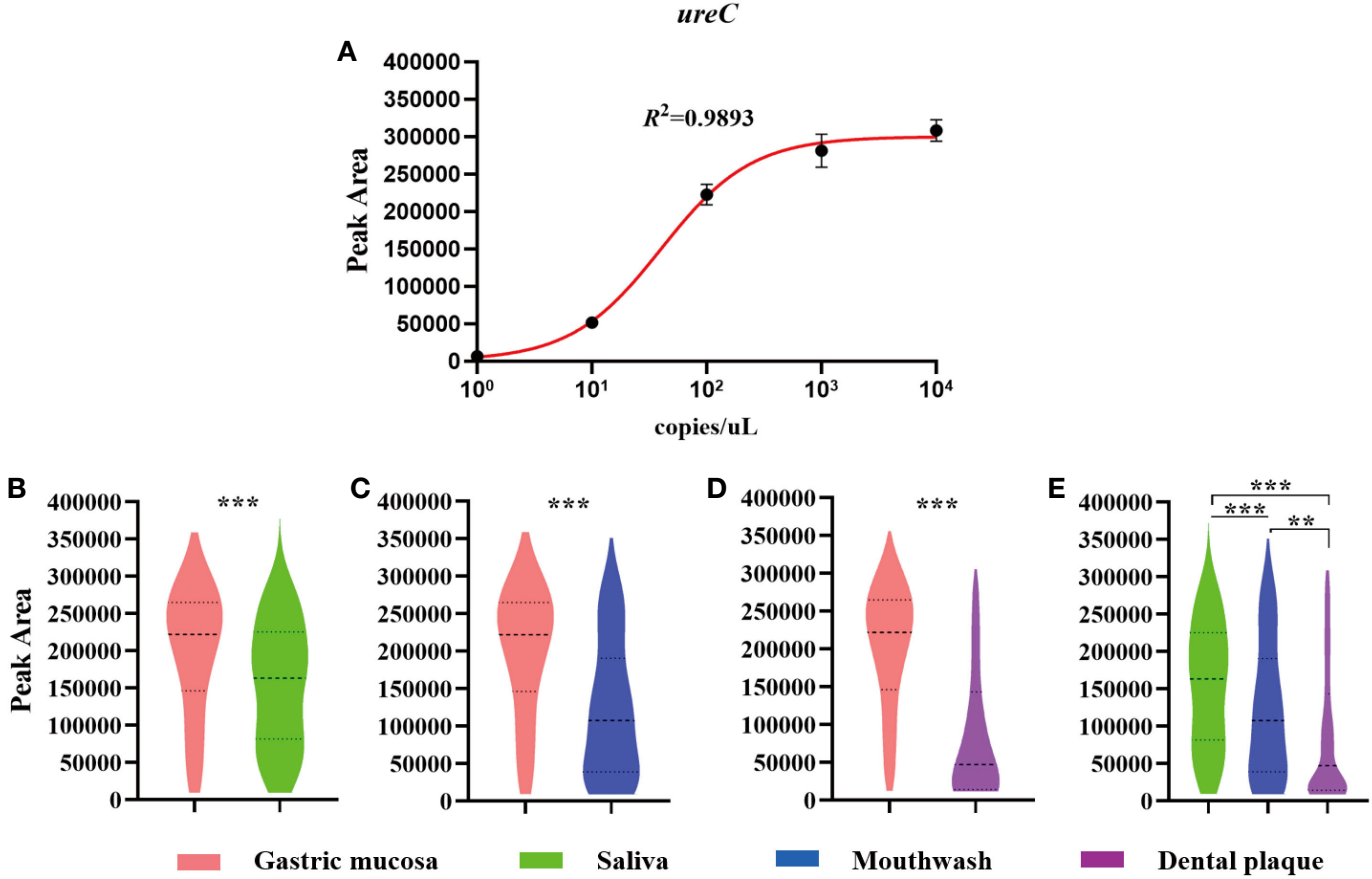
The semi-quantitation of *H. pylori* in clinical samples by detecting the peak area of *ureC* using non-invasive HMGA. The violin plot was drawn by GraphPad Prism 8.0.2. **(A)** The peak areas of *ureC* at gradient concentration of 1×10^0^~1×10^4^ copies/µL in simulated positive saliva were detected by non-invasive HMGA to evaluate the semi-quantitation for *H. pylori.* **(B–D)** The peak area levels of *ureC* in gastric mucosa samples were significantly higher than those in the three oral samples (*p*<0.001). **(E)** The peak area levels of *ureC* in saliva were significantly higher than those in mouthwash (*p<*0.001) and dental plaque (*p* < 0.001). **denotes *p* < 0.01, ***denotes *p* < 0.001.

Our data showed that the detection peak area of non-invasive HMGA increased with the increase of *ureC* concentration. The correlation coefficient between *ureC* gene concentration and peak area was 0.989 (**Figure 3A**). We further used non-invasive HMGA to detect the peak area of *ureC* gene in gastric mucosa and oral samples of 242 patients and drew violin graphs based on the distribution and probability density of peak area of *ureC* in different samples. As shown, the peak area of *ureC* in gastric mucosa samples was relatively high (from 150000 to 300000), and significantly higher than that of three oral samples (*p <*0.001) (**Figures 3B–D**). The *ureC* peak area of saliva samples was distributed in the relatively middle position (from 100 000 to 250 000), and the peak area level was significantly higher than that of mouthwash samples (*p <*0.001) and dental plaque samples (*p <*0.001) (**Figure 3E**). Furthermore, we calculated the copy numbers of *H. pylori* corresponding to the relatively concentrated detection peak area of *ureC* distribution in clinical samples. We found, more than 60% of the *ureC* peak areas of gastric mucosa concentrated around 250 000, and the corresponding copy numbers of *H. pylori* were about 100 copies/μL. More than 70% of the *ureC* peak areas of saliva samples ranged from 50 000 to 250 000, and the corresponding copy numbers of *H. pylori* were ranged from 10 to 100 copies/μL. About 50% of the *ureC* peak areas of mouthwash samples ranged from 50 000 to 250 000, and the corresponding copy numbers were ranged from 10 to 100 copies/μL. About 50% of the *ureC* peak areas of dental plaque samples below 50 000, and the corresponding to the copy numbers of *H. pylori* were less than 10 copies/μL. *H. pylori* loads in gastric mucosa (*p*<0.05) were significantly higher than those in three kinds of oral samples by detecting *ureC* peak area levels. Furthermore, according to the semi-quantitative standard analysis, when the peak areas of *H. pylori* DNA in different clinical samples concentrated around 5 000, the corresponding mean copy numbers was 10 copies/μL.

### 3.4 The non-invasive HMGA was able to distinguish different virulence genotypes

**Figure 4:**
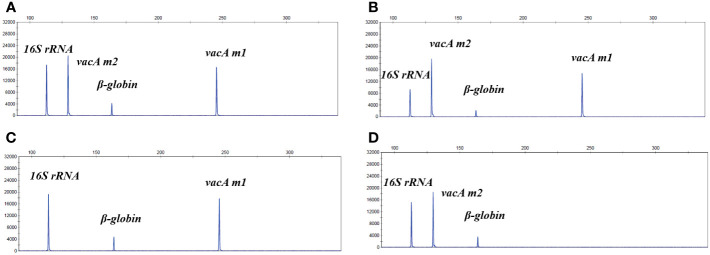
The non-invasive HMGA accurately distinguished different virulence genotypes. **(A)** The system was used to detect mixed plasmids of *vacA m*1 (10^2^ copies/μL) and *vacA m*2 (10^3^ copies/μL), and the mixed plasmids of *16S rRNA* and *β*-globin. The *vacA m*1 and *vacA m*2 were detected specific peaks of about 16000 rfu and 20000 rfu at 245 bp and 130 bp, respectively. **(B)** The system was used to detect the mixed plasmids of *vacA m*1 (10^2^ copies/μL), *vacA m*2 (10^3^ copies/μL) and negative controls (10^5^ copies/μL). **(C, D)** The position and signal intensity of the target peaks were basically consistent with those in **(A)**, when *vacA m*1 (10^2^ copies/μL) and *vacA m*2 (10^3^ copies/μL) plasmids detected separately.

To evaluate whether non-invasive HMGA could accurately identify and distinguish different *vacA* genotypes, plasmids of virulence genes *vacA m1* and *vacA m2* were mixed at final concentrations of 10^2^ and 10^3^copies/μL to form mixed templates with seven negative control pathogens DNA. As shown in **Figures 4A, B**, the position and signal intensity of the target peaks of the two virulence genes were consistent whether seven negative control pathogens DNA existed. As shown in **Figures 4C, D**, the position and signal intensity of the target peaks of the two virulence gene plasmids were basically consistent with those of the mixed detection, indicating that when non-invasive HMGA was used to detect different *vacA* genotypes, the position and signal intensity of the target peaks were not interfered.

### 3.5 Detection and clinical correlation analysis of consistency of virulence genes in oral samples by non-invasive HMGA

**Figure 5:**
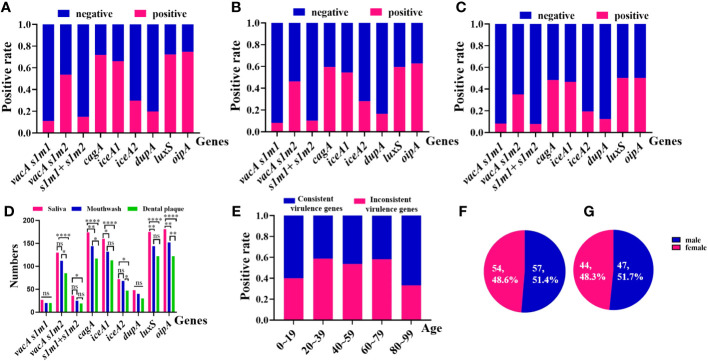
Positive rates of the consistency of virulence genes of *H. pylori* from 202 oral samples in different groups by non-invasive HMGA. **(A)** The virulence genotypes of *H. pylori* in saliva samples. From left to right were *vacA s*1*m*1** (11.2%), meaning *vacA s*1** and *vacA m1*, *vacA s*1*m*2** (53.7%), meaning *vacA s*1** and *vacA m*2**, *vacA s*1*m*1** +*vacA s*1*m*2** (14.9%), meaning *vacA s*1**, *vacA m1* and *vacA m*2**, *cagA* (71.9%), *iceA*1(66.1%), *iceA*2(29.8%), *dupA*(19.8%), *luxS*(72.3%) and *oipA*(74.8%). **(B)** The virulence genotypes of *H. pylori* in mouthwash samples. From left to right were *vacA s*1*m*1** (8.3%), *vacA s*1*m*2** (46.3%), *vacA s*1*m*1** +*vacA s*1*m*2** (10.3%), *cagA* (59.5%), *iceA*1(54.6%), *iceA*2(28.1%), *dupA*(16.5%), *luxS*(59.5%) and *oipA*(62.8%). **(C)** The virulence genotypes of *H. pylori* in dental plaque samples. From left to right *vacA s*1*m*1** (8.3%), *vacA s*1*m*2** (35.1%), *vacA s*1*m*1** +*vacA s*1*m*2** (7.9%), *cagA* (48.4%), *iceA*1(46.7%), *iceA*2(19.4%), *dupA*(12.4%), *luxS*(50.4%) and *oipA*(50.4%). **(D)** The numbers of virulence genes in saliva were higher than those in mouthwash and dental plaque, and the numbers of virulence genes in mouthwash were higher than those in dental plaque. **(E)** The distributions of the consistency of virulence genes of *H. pylori* from oral samples in different ages were compared. The proportion of consistent virulence genes of *H. pylori* in age groups 0-19, 20-39, 40-59, 60-79 and 80-99 were 40.0%, 58.8%, 53.7%, 58.3% and 33.3%, respectively. **(F)** The distributions of consistent virulence genes of *H. pylori* in different genders. **(G)** The distributions of inconsistent virulence genes of *H*. *pylori* in different genders.

Non-invasive HMGA was used to detect *H. pylori* in saliva, mouthwash and dental plaque samples of 242 patients. The results showed that *H. pylori* was positive in at least one kind of oral sample of 202 patients (83.5%, 202/242). Clinical analysis showed that the proportion of male patients with oral *H. pylori* positive was 51.5% (104/202), which was higher than that of female patients (48.5%, 98/202). Consistent with the loads of *H. pylori* in oral samples, we found that the positive detection rates of *H. pylori* virulence genotypes in saliva were higher than those in mouthwash and dental plaque. The positive detection rates of *H. pylori* virulence genotypes in mouthwash were higher than those in dental plaque. Among them, the positive detection rates of *cagA*, *iceA1*, *luxS* and *oipA* in saliva were significantly higher than those in mouthwash and dental plaque (*p <*0.05). The positive detection rates of *vacA s1m2*, *cagA*, *iceA2* and *oipA* in mouthwash were significantly higher than those in dental plaque (*p <*0.05) (**Figures 5A–D**).

By comparing the consistency of 10 virulence gene combinations of *H. pylori* in different oral samples from 202 patients, we found that in 55.0% (111/202) of patients, 10 virulence genes of *H. pylori* were completely consistent among different oral samples. And 45.0% (91/202) of patients were detected different virulence genes in different oral samples. In addition, we analyzed the epidemiological characteristics of *H. pylori* in 202 oral samples. We found that, the positive rates of consistent virulence genes of *H. pylori* were higher than those of the inconsistent virulence genes in patients aged 20-79 years old (*p*<0.05) (**Figure 5E**). The proportion of consistent virulence genes in males and females in oral samples was 51.4% (57/111) and 48.6% (54/111), and the ratio of males to females was close to 1.1:1 (**Figure 5F**). The proportion of inconsistent virulence genes in male and female patients was 51.7% (47/91) and 48.3% (44/91). The sex ratio of inconsistent virulence genes in oral samples was close to 1.1:1 (*p >*0.05) (**Figure 5G**).

### 3.6 Consistency of detecting virulence genes of *H. pylori* in oral and gastric mucosa samples by non-invasive HMGA

Firstly, we compared the consistency of 10 virulence gene combinations between saliva and gastric mucosa of each patient. The results showed that 10 virulence genes combinations in 47.5% (115/242) saliva samples were completely consistent with that in gastric mucosa, 32.6% (79/242) were different, and 19.8% (48/242) were *H. pylori* negative (**Table 4**). Secondly, we found that 10 virulence gene combinations in 38.8% (94/242) mouthwash samples were completely consistent with that in the gastric mucosa (**Supplementary Table S1**). Finally, 10 virulence gene combinations between dental plaque and gastric mucosa were completely consistent in 36.8% (89/242) patients (**Supplementary Table S2**). Furthermore, the consistency of positive detection rates of seven virulence genes (*vacA s1*, *vacA m2*, *vacA m1*, *cagA*, *oipA*, *luxS* and *dupA*) in saliva and gastric mucosa was > 84% (kappa coefficient > 0.61) (**Table 5**). The kappa values of positive detection rates of *iceA1* and *iceA2* in saliva and gastric mucosa were 0.555 and 0.426, respectively.

**Table 4:** Frequencies of virulence genes combinations of *H. pylori* from gastric mucosa and saliva specimens in 242 patients. Pink.

Virulence Genes Combinations										Stomach	Saliva		
*vacA s1*	*vacA m2*	*cagA*	*oipA*	*luxS*	*iceA1*	*dupA*	*iceA2*	*vacA m1*	*vacA s2*		C	D	N
+	+	+	+	+	+	+	+	+	-	2	2	0	0
+	+	+	+	+	+	+	+	-	-	7	4	2	1
+	+	+	+	+	+	+	-	+	-	7	7	0	0
+	+	+	+	+	+	+	-	-	-	20	9	10	1
+	+	+	+	+	+	-	-	+	-	12	9	3	0
+	+	+	+	+	+	-	-	-	-	63	29	32	2
+	+	+	+	+	+	-	+	-	-	20	13	4	3
+	+	+	+	+	+	-	+	+	-	7	6	1	0
+	+	+	+	+	-	-	+	-	-	5	2	1	2
+	+	+	+	+	-	+	-	+	-	2	0	2	0
+	+	+	+	+	-	-	-	-	-	4	2	1	1
+	+	+	+	-	+	+	-	+	-	1	0	1	0
+	+	+	+	-	+	-	+	-	-	2	2	0	0
+	+	+	+	-	-	+	-	-	-	2	2	0	0
+	+	+	-	+	+	+	-	-	-	1	0	0	1
+	+	+	-	-	-	+	-	-	-	3	1	1	1
+	+	-	+	+	+	-	-	-	-	4	4	0	0
+	+	-	+	+	-	-	+	-	-	1	1	0	0
+	+	-	-	+	+	+	-	-	-	1	1	0	0
+	+	-	+	+	+	+	-	-	-	1	1	0	0
+	-	+	+	+	+	-	+	+	-	2	1	1	0
+	-	+	-	+	-	+	-	+	-	1	1	0	0
+	-	-	+	+	+	-	-	+	-	1	1	0	0
+	-	+	+	+	+	+	-	+	-	6	3	2	1
+	-	+	+	+	+	-	-	+	-	25	9	9	7
+	-	-	-	+	+	-	-	+	-	1	1	0	0
+	-	+	+	+	-	-	-	+	-	7	4	3	0

C, Consistent genotypes between gastric mucosa and saliva specimens;

D, Distinct genotypes between gastric mucosa and saliva specimens;

N, non-invasive HMGA negative for *H. pylori* in saliva specimens. Positive for *H. pylori*; Blue, negative for *H. pylori*.

**Table 5:** Comparison of consistency between each virulence gene of *H. pylori* in saliva and gastric mucosa samples based on Kappa coefficient.

Virulence Genes	Numbers of consistency in saliva and gastric mucosa		Concordance rate	Kappa
	Positive	Negative		
*vacA s1*	188	28	89.3%	0.621
*vacA m2*	147	58	84.7%	0.646
*cagA*	171	39	86.8%	0.630
*oipA*	178	38	89.3%	0.680
*luxS*	171	38	86.4%	0.615
*iceA1*	149	48	81.4%	0.555
*dupA*	38	178	89.3%	0.677
*iceA2*	33	157	78.5%	0.426
*vacA m1*	53	158	87.2%	0.685

## 4 Discussion

### 4.1 The HMGA provided a new direction and idea for non-invasive detection of *H. pylori*

*H. pylori* is a gram-negative bacillus closely related to the occurrence and development of gastric-related diseases (Zang et al., 2023). Effective and timely detection of *H. pylori* is important to prevent and treat gastric cancer and other gastric-related diseases (Ebell, 2023). The clinical traditional detection methods of *H. pylori*, such as culture, rapid urease test, immunohistochemical staining, are all invasive methods, which seriously affect the timely diagnosis of *H. pylori* (Idowu et al., 2022). By referring to the reported high detection rates of virulence genes *oipA*, *iceA* and *dupA*, etc. (Xue et al., 2021), we developed and optimized a non-invasive HMGA directly from the clinical oral specimens. It could identify 12 targets of *H. pylori*, including identification, semi-quantification and virulence genes, directly from one oral sample within 4 hours. Synchronous detection in one tube is easy to operate, and the detection cost is low for each target ($1.3). Namely, the non-invasive HMGA was fast, convenient and cost-effective. It broke through the bottleneck of invasive sampling of conventional detection methods and made up for the limitations and deficiencies of current methods.

### 4.2 The detection performance of *H. pylori* in oral samples was reliable

The sensitivity, specificity, accuracy and consistency of the non-invasive HMGA for the detection of *H. pylori* in oral samples were all greater than 0.99, confirming that it had excellent detection performance for the identification of *H. pylori* from three kinds of oral samples. The gold standard is Sanger sequencing, and other clinical methods are used to detect *H. pylori* in parallel. The sensitivities of RUT to detect *H. pylori* gradually decreased in gastric mucosa, saliva, mouthwash and dental plaque samples, which may be corelated with the less *H. pylori* loads in oral samples than those in gastric mucosa samples (Liu et al., 2019). Considering RUT is fast, simple and easy to operate.(Dahlén et al., 2018), it was used in this study for detecting *H. pylori* in oral samples, although its results maybe doubtful due to the existence of urease producing organisms such as *Haemophilus*, *Actinomyces* and *Streptococcus* sp. present in the human oral cavity. And our study also confirmed the lower specificity of RUT in oral samples (0.51), than that in gastric mucosa (0.71). In addition, the sensitivity, specificity, accuracy and consistency of qPCR kit for *H. pylori* detection in gastric mucosa were all greater than 0.95, which showed reliable detection performance. However, the detection sensitivity of this method for *H. pylori* in oral samples decreased to about 0.87, which may be related to the fact that the kit did not specifically improve the limit detection with relatively low loads of *H. pylori* in oral samples. The specificity of culture in gastric mucosa samples was as high as 1.00, but the sensitivity was low, only 0.27, due to the harsh and time-consuming culture conditions of *H. pylori*. In addition, we tried to use three kinds of oral samples for *H. pylori* culture, but it failed due to too many colonization bacteria in the oral cavity. Furthermore, the negative results of oral samples from 30 healthy volunteers by non-invasive HMGA indicated that oral microflora did not affect the detection performance of the HMGA.

### 4.3 Non-invasive HMGA could be used to detect the consistency of virulence genes in oral samples of *H. pylori*

The inconsistent virulence genes of *H. pylori* in the oral cavity may be related to the open environment in oral cavity (Momtaz et al., 2012). Some studies have found significant differences of *H. pylori* genotypes in gastric mucosa, saliva and fecal samples, and different *H. pylori* genotypes may exist in the different site of digestive tract in the same patient (Momtaz et al., 2012; Sepúlveda et al., 2012; Wang et al., 2020). The findings suggested that *H. pylori* were not consistently in saliva and dental plaque samples, which may be the result of occasional gastroesophageal reflux (Momtaz et al., 2012; Mao et al., 2021). Some researchers have indicated that *H. pylori* in oral cavity may serve as the important source of gastric reinfection (Abdul et al., 2023). Our results of non-invasive HMGA showed 45.0% patients with inconsistent virulence genes of *H. pylori* in oral samples, which might because humans can be simultaneously infected with two or more *H. pylori* genotypes (Momtaz et al., 2010; Momtaz et al., 2012).

### 4.4 The consistency of *H. pylori* genotypes in saliva and gastric mucosa by non-invasive HMGA was high

Previous studies have shown that *H. pylori* in oral cavity was closely related to *H. pylori* in stomach (Wang et al., 2014). Eradication of *H. pylori* in oral cavity may effectively reduce the positive rate of gastric *H. pylori* infection (Wang et al., 2014). It was of great clinical significance to accurately detect and analyze the genotypes consistency of *H. pylori* in oral samples and gastric mucosa. In this study, we analyzed the consistency of 10 virulence genes of *H. pylori* in saliva, mouthwash and dental plaque, as well as that in gastric mucosa. The results showed that the combination genotypes of identification gene and 10 virulence genes in saliva and gastric mucosa was completely consistent in 47.5% (115/242) patients (**Table 4**). In addition, the consistency of each virulence gene in saliva and gastric mucosa samples was more than 0.78, which confirmed that *H. pylori* from oral samples was highly consistent with gastric mucosa. Our results were consistent with the findings reported by Wang et al, which showed 64% homology between saliva and gastric samples from the same patients (Wang et al., 2002). These findings supported the notion that saliva was a possible source of *H. pylori* infection (Momtaz et al., 2012). Based on the non-invasive HMGA, we first realized the simultaneous detection of 10 important virulence genes of *H. pylori* in oral samples and analyzed the consistency of *H. pylori* in oral samples and gastric tissues, providing a more comprehensive, detailed and accurate diagnosis basis for clinical *H. pylori* infection.

## 5 Conclusion

In this study, based on oral samples, a non-invasive HMGA was established and optimized for the identification, semi-quantitation and virulence genes of *H. pylori*. In addition, the different distribution of *H. pylori* virulence genes in oral and gastric mucosa samples of Shanghai population were analyzed by non-invasive HMGA. This study developed a new method for simple, non-invasive, rapid and accurate detection of *H. pylori*, and provided a timelier and more comprehensive diagnosis basis for *H. pylori* infection. It was expected to become an effective assay for clinical identification and monitoring of *H. pylori*. Considering the frequent update of *H. pylori* virulence genes, we would develop and simplify new versions of non-invasive HMGA system, for the better, easier and more robust diagnosis of *H. pylori* infection.

## Data availability statement

The original contributions presented in the study are included in the article/**Supplementary Material**. Further inquiries can be directed to the corresponding authors.

## Ethics statement

The studies involving humans were approved by Medical Ethics Committee of Huadong Hospital Affiliated to Fudan University. The studies were conducted in accordance with the local legislation and institutional requirements. The participants provided their written informed consent to participate in this study.

## Author contributions

WC: Writing – original draft, Writing – review & editing, Formal Analysis, Software, Data curation, Methodology. SuW: Writing – review & editing, Writing – original draft, Formal Analysis, Validation. TL: Methodology, Writing – review & editing. WJ: Data curation, Writing – review & editing. LD: Data curation, Writing – review & editing. YM: Data curation, Writing – review & editing. FY: Software, Writing – review & editing. JZ: Software, Writing – review & editing. DJ: Methodology, Writing – review & editing. ZX: Methodology, Writing – review & editing. HWZ: Resources, Writing – review & editing. YW: Resources, Writing – review & editing. ZB: Project administration, Writing – review & editing, Supervision. HuZ: Project administration, Writing – review & editing, Funding acquisition, Investigation, Validation, Supervision. ShW: Supervision, Writing – review & editing, Funding acquisition, Investigation, Project administration, Validation.

## References

[B1] Abdul N. S. Khalid Alkhelaiwi A. Awadh Alenazi A. Fehaid Alrashidi R. Ghaleb Salma R. (2023). The association of helicobacter pylori in the oral cavity with dental caries in patients with and without gastric infection: A systematic review. Cureus 15 (5), e38398. doi: 10.7759/cureus.38398 37265909 PMC10231896

[B2] Chen X. Wang N. Wang J. Liao B. Cheng L. Ren B. (2022). The interactions between oral-gut axis microbiota and Helicobacter pylori. Front. Cell Infect. Microbiol. 12. doi: 10.3389/fcimb.2022.914418 PMC9381925 35992177

[B3] Chen Y. Yang C. You N. Zhang J. (2023). Relationship between Helicobacter pylori and glycated hemoglobin: a cohort study. Front. Cell Infect. Microbiol. 13. doi: 10.3389/fcimb.2023.1196338 PMC10288807 37360526

[B4] Dahlén G. Hassan H. Blomqvist S. Carlén A. (2018). Rapid urease test (RUT) for evaluation of urease activity in oral bacteria in *vitro* and in supragingival dental plaque ex vivo. BMC Oral. Health 18 (1), 89. doi: 10.1186/s12903-018-0541-3 29776416 PMC5960132

[B5] Drevinek P. Hollweck R. Lorenz M. G. Lustig M. Bjarnsholt T. (2023). Direct 16S/18S rRNA gene PCR followed by Sanger sequencing as a clinical diagnostic tool for detection of bacterial and fungal infections: a systematic review and meta-analysis. J. Clin. Microbiol., e0033823. doi: 10.1128/jcm.00338-23 37367430 PMC10575125

[B6] Ebell M. H. (2023). H. pylori Eradication: Effective for Cure or Improvement of Functional Dyspepsia, Especially if Eradication Is Confirmed. Am. Fam Physician 107 (6), Online. 37327178

[B7] Eisdorfer I. Shalev V. Goren S. Chodick G. Muhsen K. (2018). Sex differences in urea breath test results for the diagnosis of Helicobacter pylori infection: a large cross-sectional study. Biol. Sex Differ 9 (1), 1. doi: 10.1186/s13293-017-0161-7 29291751 PMC5749022

[B8] Hu B. Zhao F. Wang S. Olszewski M. A. Bian H. Wu Y.. (2016). A high-throughput multiplex genetic detection system for Helicobacter pylori identification, virulence and resistance analysis. Future Microbiol. 11, 1261–1278. doi: 10.2217/fmb-2016-0023 27023051

[B9] Idowu S. Bertrand P. P. Walduck A. K. (2022). Gastric organoids: Advancing the study of H. pylori pathogenesis and inflammation. Helicobacter 27 (3), e12891. doi: 10.1111/hel.12891 35384141 PMC9287064

[B10] Kesharwani A. Dighe O. R. Lamture Y. (2023). Role of helicobacter pylori in gastric carcinoma: A review. Cureus 15 (4), e37205. doi: 10.7759/cureus.37205 37159779 PMC10163845

[B11] Kismat S. Tanni N. N. Akhtar R. Roy C. K. Rahman M. M. Molla M. M. A.. (2022). Diagnosis and comparison of three invasive detection methods for helicobacter pylori infection. Microbiol. Insights 15, 11786361221133947. doi: 10.1177/11786361221133947 36325107 PMC9619850

[B12] Kocsmár É. Lotz G. (2022). Comment on skrebinska et al. who could be blamed in the case of discrepant histology and serology results for helicobacter pylori detection? Diagnostics. Diagnostics (Basel) 1212 (6), 133. doi: 10.3390/diagnostics12061424 35741234 PMC9222081

[B13] Kotilea K. Iliadis E. Nguyen J. Salame A. Mahler T. Miendje Deyi V. Y.. (2023). Antibiotic resistance, heteroresistance, and eradication success of Helicobacter pylori infection in children. Helicobacter 28 (5), e13006. doi: 10.1111/hel.13006 37402147

[B14] Liu Q. Li X. Zhang Y. Song Z. Li R. Ruan H.. (2019). Orally-administered outer-membrane vesicles from Helicobacter pylori reduce H. pylori infection via Th2-biased immune responses in mice. Pathog. Dis. 77 (5), ftz050. doi: 10.1093/femspd/ftz050 31504509

[B15] Mao X. Jakubovics N. S. Bächle M. Buchalla W. Hiller K. A. Maisch T.. (2021). Colonization of Helicobacter pylori in the oral cavity - an endless controversy? Crit. Rev. Microbiol. 47 (5), 612–629. doi: 10.1080/1040841x.2021.1907740 33899666

[B16] McNulty C. A. M. (2023). The first 5 years of Helicobacter pylori research-With an emphasis on the United Kingdom. Helicobacter 28 (4), e12982. doi: 10.1111/hel.12982 37102496

[B17] Momtaz H. Souod N. Dabiri H. (2010). Comparison of the virulence factors of Helicobacter pylori isolated in stomach and saliva in Iran. Am. J. Med. Sci. 340 (5), 345–349. doi: 10.1097/MAJ.0b013e3181d94fbc 21048436

[B18] Momtaz H. Souod N. Dabiri H. Sarshar M. (2012). Study of Helicobacter pylori genotype status in saliva, dental plaques, stool and gastric biopsy samples. World J. Gastroenterol. 18 (17), 2105–2111. doi: 10.3748/wjg.v18.i17.2105 22563199 PMC3342610

[B19] Nagata R. Sato H. Takenaka S. Yokoyama J. Terai S. Mimuro H.. (2023). Analysis of genetic relatedness between gastric and oral helicobacter pylori in patients with early gastric cancer using multilocus sequence typing. Int. J. Mol. Sci. 24 (3), 2211. doi: 10.3390/ijms24032211 36768541 PMC9917182

[B20] Sepúlveda E. Moreno J. Spencer M. L. Quilodrán S. Brethauer U. Briceño C.. (2012). [Comparison of Helicobacter pylori in oral cavity and gastric mucosa according to virulence genotype (cagA and vacA m 1)]. Rev. Chil. Infectol 29 (3), 278–283. doi: 10.4067/s0716-10182012000300005 23096467

[B21] Siavoshi F. Salmanian A. H. Akbari F. Malekzadeh R. Massarrat S. (2005). Detection of Helicobacter pylori-specific genes in the oral yeast. Helicobacter 10 (4), 318–322. doi: 10.1111/j.1523-5378.2005.00319.x 16104948

[B22] Siavoshi F. Saniee P. Khalili-Samani S. Hosseini F. Malakutikhah F. Mamivand M.. (2015). Evaluation of methods for H. pylori detection in PPI consumption using culture, rapid urease test and smear examination. Ann. Transl. Med. 3 (1), 11. doi: 10.3978/j.issn.2305-5839.2014.11.16 25705643 PMC4293475

[B23] Sohrabi A. Franzen J. Tertipis N. Zagai U. Li W. Zheng Z.. (2021). Efficacy of loop-mediated isothermal amplification for H. pylori detection as point-of-care testing by noninvasive sampling. Diagnostics (Basel) 11 (9), 1538. doi: 10.3390/diagnostics11091538 34573879 PMC8467764

[B24] Sun Z. Liu W. Zhang J. Wang S. Yang F. Fang Y.. (2021). The direct semi-quantitative detection of 18 pathogens and simultaneous screening for nine resistance genes in clinical urine samples by a high-throughput multiplex genetic detection system. Front. Cell Infect. Microbiol. 11. doi: 10.3389/fcimb.2021.660461 PMC8072482 33912478

[B25] Wang J. Chi D. S. Laffan J. J. Li C. Ferguson D. A. Jr. Litchfield P.. (2002). Comparison of cytotoxin genotypes of Helicobacter pylori in stomach and saliva. Dig Dis. Sci. 47 (8), 1850–1856. doi: 10.1023/a:1016417200611 12184541

[B27] Wang Y. H. Wang F. F. Gong X. L. Yan L. L. Zhao Q. Y. Song Y. P.. (2020). Genotype profiles of Helicobacter pylori from gastric biopsies and strains with antimicrobial-induced resistance. Therap Adv. Gastroenterol. 13, 1756284820952596. doi: 10.1177/1756284820952596 PMC7522827 33029198

[B26] Wang X. M. Yee K. C. Hazeki-Taylor N. Li J. Fu H. Y. Huang M. L.. (2014). Oral Helicobacter pylori, its relationship to successful eradication of gastric H. pylori and saliva culture confirmation. J. Physiol. Pharmacol. 65 (4), 559–566. 25179088

[B28] Xu Y. Song Y. Wang X. Gao X. Li S. Yee J. K. (2018). A clinical trial on oral H. pylori infection of preschool children. Ann. Clin. Lab. Sci. 48 (6), 751–756. 30610045

[B29] Xue Z. Yang H. Su D. Song X. Deng X. Yu C.. (2021). Geographic distribution of the cagA, vacA, iceA, oipA and dupA genes of Helicobacter pylori strains isolated in China. Gut Pathog. 13 (1), 39. doi: 10.1186/s13099-021-00434-4 34130751 PMC8207754

[B30] Zang H. Wang J. Wang H. Guo J. Li Y. Zhao Y.. (2023). Metabolic alterations in patients with Helicobacter pylori-related gastritis: The H. pylori-gut microbiota-metabolism axis in progression of the chronic inflammation in the gastric mucosa. Helicobacter 28 (4), e12984. doi: 10.1111/hel.12984 37186092

[B31] Zhang Y. Wang S. Hu B. Zhao F. Xiang P. Ji D.. (2016a). Direct detection of Helicobacter pylori in biopsy specimens using a high-throughput multiple genetic detection system. Future Microbiol. 11, 1521–1534. doi: 10.2217/fmb-2016-0149 27599152 PMC5562010

[B32] Zhang Y. Zhao F. Kong M. Wang S. Nan L. Hu B.. (2016b). Validation of a high-throughput multiplex genetic detection system for helicobacter pylori identification, quantification, virulence, and resistance analysis. Front. Microbiol. 7. doi: 10.3389/fmicb.2016.01401 PMC5013035 27656172

[B33] Zhou L. Zhao F. Hu B. Fang Y. Miao Y. Huang Y.. (2016). A creative helicobacter pylori diagnosis scheme based on multiple genetic analysis system: qualification and quantitation. Helicobacter 21 (5), 441. doi: 10.1111/hel.12352 27621151

