# Supplementary material for: A rapid and high-throughput multiplex genetic detection assay for detection, semi-quantification and virulence genotyping of Helicobacter pylori in non-invasive oral samples
Source: Front Cell Infect Microbiol. 2023 Sep 29;13:1267288. doi: 10.3389/fcimb.2023.1267288 (PMC10570550; doi:10.3389/fcimb.2023.1267288)
Supplement: Supplementary file 1 [file DataSheet_1.docx]

Supplementary Material

# Supplementary Figures and Tables

# 1.1 Supplementary Figures

Supplementary Figure S1

| 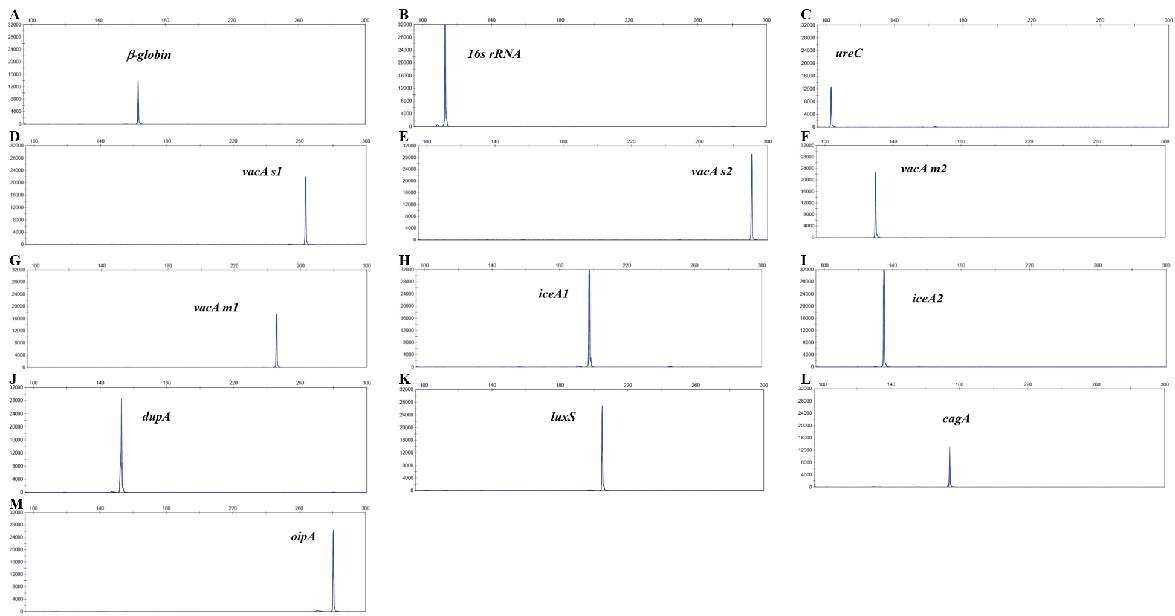 |
| --- |

Supplementary Figure S1 The non-invasive HMGA could produce specific amplification signals for all targets with plasmids. The horizontal coordinate indicated the actual PCR product size(bp), and the vertical coordinate indicated the dye signal (rfu). (A-M) The amplicon sizes for the target genes were as follows: *ureC* (103 bp), *16S rRNA* (114 bp), *vacA m*2 (130bp), *iceA2* (135 bp), *dupA* (152 bp), *β-globin* (164 bp), *cagA* (174 bp), *iceA1* (198 bp), *luxS* (205 bp), *vacA m*1 (245 bp), *vacA s*1 (263 bp), *oipA* (281 bp) and *vacA s*2 (290 bp).

Supplementary Figure S2

| 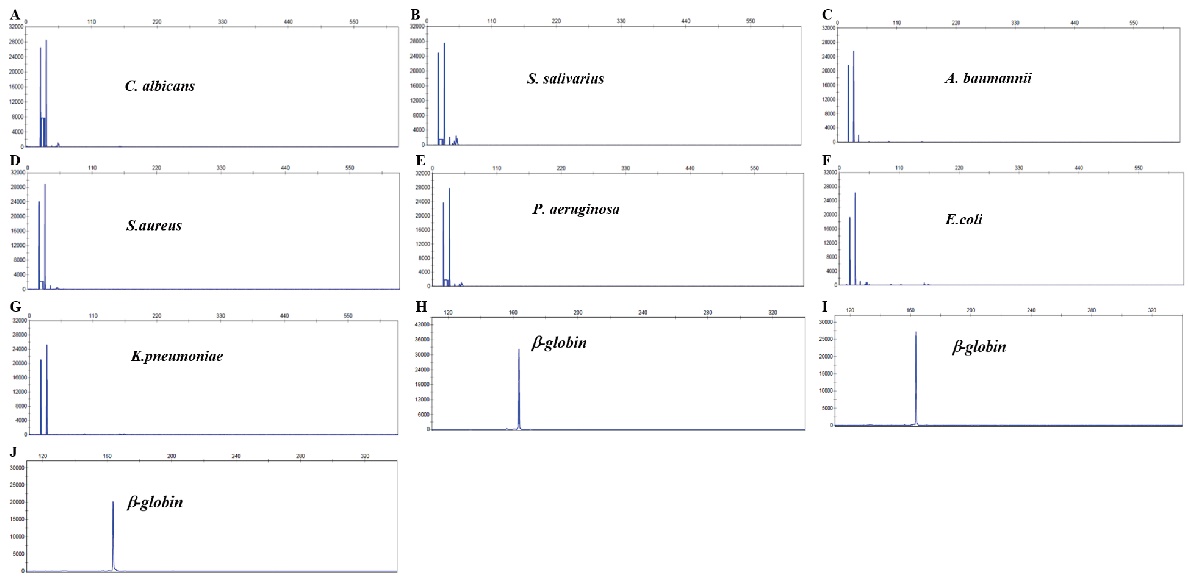 |
| --- |

Supplementary Figure S2 Negative control pathogens and *H. pylori* negative oral samples did not affect the specificity of non-invasive HMGA. The horizontal coordinate indicated the actual PCR product size(bp), and the vertical coordinate indicated the dye signal (rfu). (A-G) Negative control pathogens, including *C. albicans*, *S. salivarius*, *A. baumannii*, *S.aureus*, *P. aeruginosa*, *E.coli* and *K. pneumoniae* did not produce any specific amplification peaks. (H-J) All *H. pylori* negative oral samples did not produce any specific amplification peaks.

## 1.2 Supplementary Tables

Supplementary Table S1 Frequencies of virulence genes combinations of *H. pylori* from gastric mucosa and mouthwash specimens in 242 patients

| **Virulence Genes Combinations** | | | | | | | | | | **Stomach** | **mouthwash** | | |
| --- | --- | --- | --- | --- | --- | --- | --- | --- | --- | --- | --- | --- | --- |
| ***vacA s1*** | ***vacA m2*** | ***cagA*** | ***oipA*** | ***luxS*** | ***iceA1*** | ***dupA*** | ***iceA2*** | ***vacA m1*** | ***vacA s2*** |  | **C** | **D** | **N** |
| + | + | + | + | + | + | + | + | + | - | 2 | 1 | 0 | 1 |
| + | + | + | + | + | + | + | + | - | - | 7 | 4 | 2 | 1 |
| + | + | + | + | + | + | + | - | + | - | 7 | 5 | 1 | 1 |
| + | + | + | + | + | + | + | - | - | - | 20 | 7 | 9 | 4 |
| + | + | + | + | + | + | - | - | + | - | 12 | 9 | 3 | 0 |
| + | + | + | + | + | + | - | - | - | - | 63 | 29 | 21 | 13 |
| + | + | + | + | + | + | - | + | - | - | 20 | 11 | 2 | 7 |
| + | + | + | + | + | + | - | + | + | - | 7 | 3 | 3 | 1 |
| + | + | + | + | + | - | - | + | - | - | 5 | 1 | 3 | 1 |
| + | + | + | + | + | - | + | - | + | - | 2 | 2 | 0 | 0 |
| + | + | + | + | + | - | - | - | - | - | 4 | 0 | 4 | 0 |
| + | + | + | + | - | + | + | - | + | - | 1 | 0 | 1 | 0 |
| + | + | + | + | - | + | - | + | - | - | 2 | 1 | 0 | 1 |
| + | + | + | + | - | - | + | - | - | - | 2 | 1 | 0 | 1 |
| + | + | + | - | + | + | + | - | - | - | 1 | 1 | 0 | 0 |
| + | + | + | - | - | - | + | - | - | - | 3 | 1 | 1 | 1 |
| + | + | - | + | + | + | - | - | - | - | 4 | 2 | 1 | 1 |
| + | + | - | + | + | - | - | + | - | - | 1 | 0 | 0 | 1 |
| + | + | - | - | + | + | + | - | - | - | 1 | 1 | 0 | 0 |
| + | + | - | + | + | + | + | - | - | - | 1 | 1 | 0 | 0 |
| + | - | + | + | + | + | - | + | + | - | 2 | 1 | 0 | 1 |
| + | - | + | - | + | - | + | - | + | - | 1 | 0 | 1 | 0 |
| + | - | - | + | + | + | - | - | + | - | 1 | 0 | 0 | 1 |
| + | - | + | + | + | + | + | - | + | - | 6 | 3 | 1 | 2 |
| + | - | + | + | + | + | - | - | + | - | 25 | 6 | 8 | 11 |
| + | - | - | - | + | + | - | - | + | - | 1 | 1 | 0 | 0 |
| + | - | + | + | + | - | - | - | + | - | 7 | 3 | 2 | 2 |

**C**: Consistent genotypes between gastric mucosa and mouthwash specimens;

**D**: Distinct genotypes between gastric mucosa and mouthwash specimens;

**N**: non-invasive HMGA system negative for *H. pylori* in mouthwash specimens.

**Pink**, positive for *H. pylori*; **Blue**, negative for *H. pylori.*

Supplementary Table S2. Frequencies of virulence genes combinations of *H. pylori* from gastric mucosa and dental plaque specimens in 242 patients

| **Virulence Genes Combinations** | | | | | | | | | | **Stomach** | **dental plaque** | | |
| --- | --- | --- | --- | --- | --- | --- | --- | --- | --- | --- | --- | --- | --- |
| ***vacA s1*** | ***vacA m2*** | ***cagA*** | ***oipA*** | ***luxS*** | ***iceA1*** | ***dupA*** | ***iceA2*** | ***vacA m1*** | ***vacA s2*** |  | **C** | **D** | **N** |
| + | + | + | + | + | + | + | + | + | - | 2 | 1 | 1 | 0 |
| + | + | + | + | + | + | + | + | - | - | 7 | 3 | 2 | 2 |
| + | + | + | + | + | + | + | - | + | - | 7 | 3 | 1 | 3 |
| + | + | + | + | + | + | + | - | - | - | 20 | 10 | 3 | 7 |
| + | + | + | + | + | + | - | - | + | - | 12 | 8 | 2 | 2 |
| + | + | + | + | + | + | - | - | - | - | 63 | 30 | 13 | 20 |
| + | + | + | + | + | + | - | + | - | - | 20 | 7 | 2 | 11 |
| + | + | + | + | + | + | - | + | + | - | 7 | 5 | 1 | 1 |
| + | + | + | + | + | - | - | + | - | - | 5 | 1 | 0 | 4 |
| + | + | + | + | + | - | + | - | + | - | 2 | 1 | 1 | 0 |
| + | + | + | + | + | - | - | - | - | - | 4 | 0 | 1 | 3 |
| + | + | + | + | - | + | + | - | + | - | 1 | 0 | 0 | 1 |
| + | + | + | + | - | + | - | + | - | - | 2 | 0 | 0 | 2 |
| + | + | + | + | - | - | + | - | - | - | 2 | 1 | 0 | 1 |
| + | + | + | - | + | + | + | - | - | - | 1 | 1 | 0 | 0 |
| + | + | + | - | - | - | + | - | - | - | 3 | 0 | 1 | 2 |
| + | + | - | + | + | + | - | - | - | - | 4 | 2 | 1 | 1 |
| + | + | - | + | + | - | - | + | - | - | 1 | 0 | 0 | 1 |
| + | + | - | - | + | + | + | - | - | - | 1 | 1 | 0 | 0 |
| + | + | - | + | + | + | + | - | - | - | 1 | 1 | 0 | 0 |
| + | - | + | + | + | + | - | + | + | - | 2 | 1 | 0 | 1 |
| + | - | + | - | + | - | + | - | + | - | 1 | 0 | 0 | 1 |
| + | - | - | + | + | + | - | - | + | - | 1 | 0 | 0 | 1 |
| + | - | + | + | + | + | + | - | + | - | 6 | 3 | 0 | 3 |
| + | - | + | + | + | + | - | - | + | - | 25 | 6 | 14 | 5 |
| + | - | - | - | + | + | - | - | + | - | 1 | 1 | 0 | 0 |
| + | - | + | + | + | - | - | - | + | - | 7 | 3 | 2 | 2 |

**C**: Consistent genotypes between gastric mucosa and dental plaque specimens;

**D**: Distinct genotypes between gastric mucosa and dental plaque specimens;

**N**: non-invasive HMGA system negative for *H. pylori* in dental plaque specimens.

**Pink**, positive for *H. pylori*; **Blue**, negative for *H. pylori.*
